# HBV DNA Integration: Molecular Mechanisms and Clinical Implications

**DOI:** 10.3390/v9040075

**Published:** 2017-04-10

**Authors:** Thomas Tu, Magdalena A. Budzinska, Nicholas A. Shackel, Stephan Urban

**Affiliations:** 1 Department of Infectious Diseases, Molecular Virology, Heidelberg University, Im Neuenheimer Feld 345, 69120 Heidelberg, Germany; Stephan.Urban@med.uni-heidelberg.de; 2 Centenary Institute, The University of Sydney, Sydney, NSW 2050, Australia; m.budzinska@centenary.org.au (M.A.B.); n.shackel@unsw.edu.au (N.A.S.); 3 Sydney Medical School, The University of Sydney, Sydney, NSW 2006, Australia; 4 Liverpool Hospital, Gastroenterology, Sydney, NSW 2170, Australia; 5 German Center for Infection Research (DZIF), Heidelberg Partner Site, Im Neuenheimer Feld 345, 69120 Heidelberg, Germany

**Keywords:** hepatitis B virus, integration, hepatocellular carcinoma, non-homologous end joining, viral persistence

## Abstract

Chronic infection with the Hepatitis B Virus (HBV) is a major cause of liver-related morbidity and mortality. One peculiar observation in cells infected with HBV (or with closely‑related animal hepadnaviruses) is the presence of viral DNA integration in the host cell genome, despite this form being a replicative dead-end for the virus. The frequent finding of somatic integration of viral DNA suggests an evolutionary benefit for the virus; however, the mechanism of integration, its functions, and the clinical implications remain unknown. Here we review the current body of knowledge of HBV DNA integration, with particular focus on the molecular mechanisms and its clinical implications (including the possible consequences of replication-independent antigen expression and its possible role in hepatocellular carcinoma). HBV DNA integration is likely to influence HBV replication, persistence, and pathogenesis, and so deserves greater attention in future studies.

## 1. Introduction

### 1.1. Hepatitis B Virus Infection

Infection with the human hepatitis B virus (HBV) is one of the most widespread causes of liver cirrhosis and primary liver cancer (hepatocellular carcinoma; HCC). While a prophylactic vaccine is available to prevent HBV infection, there is currently no cure for patients with chronic hepatitis B (CHB). Chronic HBV infection currently affects approximately 240 million people worldwide, and is the main contributor to viral hepatitis-associated morbidity and mortality, which accounts for up to 1.5 million deaths—the seventh leading worldwide cause of death—and a loss of 42 million disability-adjusted life years (DALYs) annually [1].

Upon initial exposure to HBV, the failure to induce a significant innate immune response within hepatocytes and the immunosuppressive liver microenvironment can lead to incomplete clearance of infected hepatocytes and the establishment of a chronic infection [2,3,4]. The natural history of CHB can be categorised into five clinical phases [5,6]: (1) immune tolerance; (2) immune reactive HBV e antigen (HBeAg)-positive; (3) inactive HBV carrier; (4) HBeAg-negative chronic hepatitis; and (5) HBV surface antigen (HBsAg)-negative phases. A chronic HBV infection begins with the generally-asymptomatic non-inflammatory (or immune tolerant) phase. This state is characterised by high HBV serum titres (~2 × 10^9^ International Units (IU) per mL) and poorly-activated (but not completely silent [7]) HBV-specific CD8^+^ T-cells [8,9]. The quality of the anti-HBV immunological state (whether truly tolerant, poorly activated, or absent) during this phase is currently a point of controversy [7,10,11,12,13]. After decades of chronic HBV infection in this phase, HBV-specific CD8^+^ T‑cells somehow become increasingly activated, and the infection progresses to an inflammatory (or immune reactive) HBeAg-positive phase, characterised by observable flares of immune-mediated liver damage and fluctuations in HBV titres [8,14]. Seroconversion to an anti-HBeAg-positive state and the elimination of many (but not all) HBV-infected hepatocytes heralds the entry into the inactive HBV carrier phase. In this phase, low HBV titres are observed and liver injury progression is slower compared to the preceding immune reactive phase [15,16]. Reactivation of virus replication can occur in this phase, driving chronic inflammation with flares of virological breakthrough which defines the HBeAg-negative chronic hepatitis phase [5,6]. A minority of individuals (~1% of HBeAg-negative patients per year [17]) with CHB clear the virus and progress to the HBsAg-negative phase, in which HBV serum DNA is undetectable and antibodies recognizing the HBV surface antigen can be (but are not necessarily) found. This state defines a “functional cure”, and disease progression is halted. However, reactivation (e.g., under immune suppressive therapy) is observed, indicating that the viral covalently closed circular DNA (cccDNA) remains present in the liver, but is transcriptionally silent. In general, the HBV-related end-stage liver disease (including cirrhosis and HCC) occurs decades post-exposure [18].

### 1.2. HBV Structure and Replication Cycle

**Figure 1:**
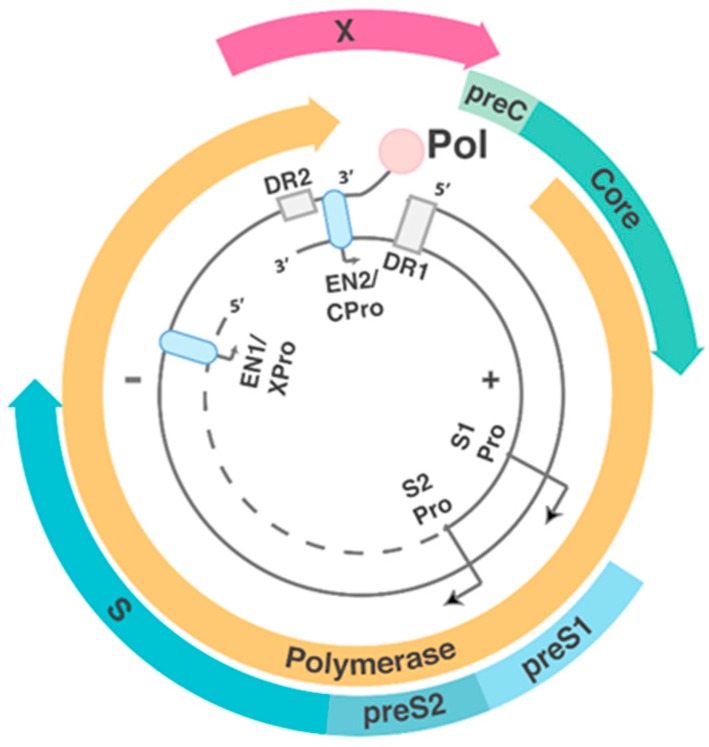
Open reading frames (ORFs) of hepatitis B virus (HBV) circular DNA form. Coloured blocks represent each of the four main open reading frames, and the associated promoters (Pro) and enhancers (EN1 and EN2, light blue ovals) are shown. The direct repeat regions (DR1 and DR2, grey boxes) play important roles in hepatitis B virus (HBV) replication (seen in Figure 2). The large, medium and small forms of HBsAg are translated from the preS1 + preS2 + S, preS2 + S and S alone ORF, respectively. Pol is encoded by the polymerase ORF. HBV core antigen (HBcAg) is translated from the core ORF alone. HBV e antigen (HBeAg) is formed by the cleavage of the translated product from PreC + core ORF. HBV X protein (HBx) is translated from the X ORF.

HBV is the prototypic member of the *Hepadnavirdae* family, composed of enveloped viruses that contain ~3.2 kbp relaxed circular double-stranded DNA (dsDNA) genomes encapsidated within virally-encoded capsids. Like other members of the *Hepadnaviridae* (e.g., duck hepatitis B virus or woodchuck hepatitis B virus), the small genome exhibits high informational density with extensively overlapping open reading frames (ORFs), with every base coding for at least one ORF. The genome contains four genes, which result in different RNAs with a common poly-A site, coding for seven different proteins (Figure 1). These include: the structural proteins, HBsAg (of which there are three forms: large, medium, and small) and HBV core antigen (HBcAg); HBV e antigen (HBeAg), a processed and secreted form of the gene product of the preCore/Core ORF; the HBV polymerase (pol); and the transcriptional transactivator HBV X protein (HBx), which controls HBV transcription from cccDNA.

**Figure 2:**
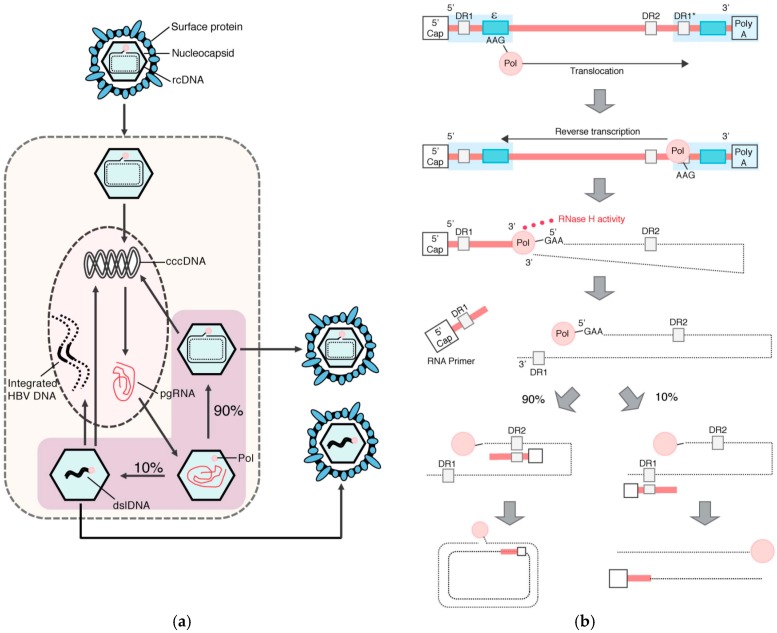
The replication cycle of HBV (**a**) with a focus on the steps of intra-capsid reverse transcription (**b**); (**a**) After the virus undergoes receptor-mediated entry via heparan sulphate proteoglycans and sodium taurocholate cotransporting polypeptide (NTCP), the nucleocapsid is transported to the nucleus, where the relaxed circular DNA (rcDNA) genome is converted into covalently closed circular (ccc)DNA by the host proteins. Covalently closed circular DNA (cccDNA) acts as the viral template for messenger RNAs (mRNA) and pregenomic RNA (pgRNA; **red line**), which is encapsidated together with the viral polymerase (**red circle**) that mediates reverse transcription (**purple area**); (**b**) During reverse transcription, Pol has three distinct functions: primer synthesis, RNA/DNA-dependent DNA polymerisation, and RNaseH-mediated RNA degradation. pgRNA is greater than genome length and thus contains redundant regions (**light blue shading**). After binding to the 5’-epsilon (ε) region of the pgRNA (**left blue box**), Pol synthesises a three nucleotide (nt) oligonucleotide (GAA), using ε as a template. The trinucleotide primer is covalently attached to the Pol, and this complex then translocates to the direct repeat 1* region (DR1*), located in the 3’region. Pol reverse transcribes a negative-sense DNA strand (**black**) using the 3 nt oligonucleotide as a primer and the pgRNA as a template. Simultaneously with reverse transcription, Pol hydrolyses the RNA template with its ribonuclease H (RNaseH) activity lagging 18 nt behind the site of reverse transcription. Since Pol is covalently attached to the 5’ end of the synthesised negative-sense DNA strand, a loop structure forms. The pgRNA is hydrolysed up to 18 nt from the 5’ end. The remaining 18 nt RNA oligonucleotide acts as a primer for the synthesis of the positive-sense DNA strand. In ~90% of nucleocapsids, the 18 nt RNA primer translocates to the DR2 sequence, leading to the synthesis of rcDNA. In the remaining 10%, the RNA primer remains bound to the DR1 region, priming double stranded linear DNA (dslDNA) synthesis. After reverse transcription, the mature nucleocapsids can either be secreted as virions or cycle to the nucleus to add to the cccDNA pool. dslDNA can also integrate into the host cell genome.

The HBV replication cycle (Figure 2a) starts with attachment and entry into hepatocytes, the main cell type of the liver. HBV initially attaches via low-specificity interactions between HBsAg within the virus envelope and heparan sulphate proteoglycans on the surface of hepatocytes [19,20,21]. A high specificity interaction then occurs between the N-terminal 75 amino acids of the preS1-domain of the large HBsAg and sodium taurocholate cotransporting polypeptide (NTCP), a hepatocyte‑specific bile salt transporter and cellular receptor for HBV [22,23].

After receptor-mediated entry of the virion, the nucleocapsid containing the relaxed circular DNA (rcDNA) genome is released into the cytoplasm and transported to the nucleus [24]. Using nuclear host cell factors involving components of the DNA repair machinery (such as TDP2 and Pol‑K [25,26]), the liberated rcDNA is converted into covalently closed circular DNA (cccDNA), the stable episomal transcriptional template for HBV messenger RNAs (mRNAs) [27]. Both subgenomic mRNAs encoding the structural and regulatory proteins, and greater-than-genome-length pregenomic RNA (pgRNA) are transcribed from cccDNA. pgRNA, along with the viral polymerase, is encapsidated into viral capsids [24]. Reverse transcription of the pgRNA occurs within the nucleocapsid via a complicated series of steps (Figure 2b), resulting in rcDNA or double-stranded linear DNA (dslDNA) forms. Throughout this process, many replicative intermediates (including non-enveloped HBV DNA-containing nucleocapsids and enveloped pgRNA-containing nucleocapsids [28,29]) are formed, and in some cases, secreted. A state-of-the-art review of these minor forms has been included as part of this special issue, highlighting in particular their still-unknown function(s) [30].

The major form of virus produced by HBV-infected cells is rcDNA-containing nucleocapsids. These can either (1) be enveloped and secreted as virions (the default pathway required for viral spread); or (2) cycle back to the nucleus, at a currently unknown rate, to add or replenish the intranuclear cccDNA pool [24].

**Figure 3:**
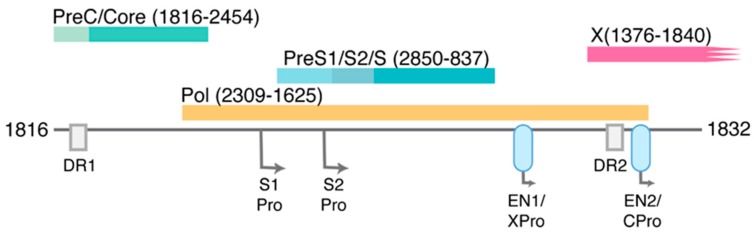
ORFs of HBV dslDNA form. As with Figure 1, coloured blocks represent each of the four main open reading frames. Associated promoters (Pro), enhancers (EN1 and EN2, light blue ovals), and direct repeat regions (DR1 and DR2) are also depicted. The approximate nts of the 5’ and 3’ ends are shown (numbering based on HBV DNA sequence from Genbank Accession #AB241115). As a result of the dslDNA forms generated by in situ priming (Figure 2b), the X ORF is truncated at its C‑terminus (by at least three amino acids) and the pre-core/core promoter is separated from its ORF.

In a minority of nucleocapsids, RNA primer translocation does not occur and reverse transcription is primed from DR1 (Figure 2b), resulting in the formation of dslDNA (Figure 3). In vitro, HBV dslDNA genomes are produced by reverse transcription within ~30% of mature nucleocapsids [31], though (for unknown reasons) the mean figure in patient sera is ~7% (ranging from 3% to 36%) [32]. The RNA primer translocation efficiency (and therefore ratio of rcDNA:dslDNA) is known to depend on both *cis*-circularisation sequences in the negative-sense HBV DNA strand as well as sequence complementarity between the DR2 and RNA primer [33,34]. Interestingly, the frequency of RNA primer translocation is sub-maximal and can be increased with small (3–5 nucleotide; nt) substitution mutations proximal to the DR2 region [34]. This suggests that there is evolutionary pressure for dslDNA to be formed.

dslDNA (like rcDNA) can be transported to the nucleus to form cccDNA (containing a redundant 16 nt insertion) or be released as virions containing dslDNA [35]. The cccDNA produced from dslDNA contains insertions that are not supportive of rcDNA synthesis, but can make up a large fraction of the cccDNA pool (up to 20% in the woodchuck hepatitis B virus model), and some forms may generate progeny dslDNA [35,36,37]. Further, dslDNA-derived cccDNA can revert back into wild-type cccDNA, possibly via homologous recombination [35].

An additional possible fate for intranuclear dslDNA genomes is integration into the host cell genome, occurring in 1 in ~10^5^–10^6^ infected cells [38,39]. dslDNA is the presumed form that integrates as virus-cell DNA junctions match up with the termini of the dslDNA form in model systems and primary tissues [39,40,41,42].

## 2. HBV DNA Integration

### 2.1. Molecular Aspects of HBV DNA Integration

HBV DNA integration occurs throughout the host genome at dsDNA breaks, with terminal deletions of up to 200 bp from the integrated HBV DNA being common [39,40,41,42]. In vitro studies in the duck HBV infection model show that integration preferentially occurs at the site of double-stranded breaks in the cell genome (artificially-induced using a recombinant restriction enzyme) [40]. HBV DNA integration in non-tumour tissues show dispersed distribution across the whole cellular genome with no specific chromosomal hot-spots or common recurring sites between patients, though tumour tissues show some enrichment in particular genomic sites (discussed below) [43,44]. Whether HBV DNA integration occurs through canonical sequence-independent non‑homologous end joining (NHEJ) or microhomology-mediated end joining (MMEJ, also known as non-classical or alternative NHEJ) [45] is still unclear, though recent studies using next-generation sequencing (NGS) suggest the latter [44].

The structural arrangement of the integrated HBV DNA form necessarily affects the expression of all viral open reading frames except the HBsAg ORF, which can stay intact and maintain its position under its native promoter (Figure 3). However, the transcription of HBsAg in the integrated form may be under altered regulation due to contextual differences between the integrated and episomal cccDNA forms. As the dslDNA form is only ~16 nt longer than genome length, integrated HBV DNA is unable to produce pgRNA, and thereby represents a replicative dead-end for the virus.

While remaining intact, the pol and HBe/HBcAg ORFs are separated from their promoters in the integrated form. Indeed, the first descriptions of the HCC-derived cell line PLC/PRF/5 that contains four integrated HBV genomes, show that only surface and not core encoding transcripts are expressed from integrated HBV DNA [46,47]. However, other HCCs have been found to express transcripts containing the HBe/HBcAg ORFs, presumably due to active cellular promoters upstream of the integration site [48]. The proportion of integrates that express HBe/HBcAg and those that are deficient remains unknown.

Finally, enhancer 1 is known to be active in the integrated form, and so can produce transcripts of the HBx ORF [49,50]. In the integrated form, the 154 amino acid (aa) HBx is likely to be truncated by at least three amino acids (Figure 3), but studies have shown that HBx C-terminal truncation mutants up to 14 aa are still functional in transcriptional transactivation [51]. Due to the lack of a stop codon in the integrated form, the 3’ end of the HBx ORF runs out the cellular DNA can produce HBV‑cellular fusion transcripts (e.g., HBx- long interspersed nuclear element 1 (LINE1) or HBx-‑mixed-lineage leukaemia 4 (MLL4) fusion transcripts that are described below). The contribution of these fusion products in HCC development is presently being investigated, and is discussed further below.

Through cloning and characterisation of HBV integrated sequences in primary tissues (from both tumour and non-tumour tissues), multiple sometimes highly-complex rearrangements of and deletions within the HBV genome were found [50,52,53,54,55,56]. Whether these rearrangements occur pre-integration (e.g., as integration of defective HBV genomes or HBV spliced variants), post-integration (e.g., via chromosomal instability), or a combination of both is not known. 

Virus DNA integration into the host chromosome is a common feature of the *Hepadnaviridae* family (observed in both orthohepadnaviruses [42,57,58] and avihepadnaviruses [39,59]). Given the highly parsimonious nature of these viruses (as shown by their characteristic small genomes and overlapping open reading frames), it seems unlikely that integration decreases the overall evolutionary replicative fitness of HBV. This must be reconciled with the fact that dslDNA in its integrated form is replication-incompetent. Therefore, the roles and clinical implications of HBV DNA integration remain an intriguing mystery in HBV biology. Here, we summarise the state-of-the-art of the field and propose possible functions of HBV integration. 

### 2.2. The Role of HBV Integration in Viral Infection

HBV DNA integration appears to occur early in infection. HBV DNA integration has been generally described in both HCC and cirrhotic patients with long-term CHB [42,46,60,61,62,63,64,65,66,67,68,69], but recently also prior to histologically observable liver damage in CHB patients [7,70]. Even earlier integration of HBV DNA has been reported in congenitally-infected children (as young as 5 months) with severe liver disease [71,72] and in patients with acute HBV infection [73]. These observations are consistent with the woodchuck and duck animal models, where integration has been observed days post-infection (dpi) with their respective viruses [38,39]. In addition to these in vivo observations, our group has evidence that integration occurs even in in vitro infection models (including HepaRG and HepG2-NTCP systems) within five days of infection (data not shown). The early nature of this phenomenon is circumstantial evidence that HBV integration is involved in the modulation of HBV replication or persistence, but the mechanism remains to be investigated in more detail.

Of particular importance are the potential clinical consequences of these integrated forms (e.g., expression of HBx as a factor overcoming silencing of cccDNA or HBsAg expression as a possible modulator of the adaptive immune system). Expression from integrated HBV DNA is likely to be regulated by different mechanisms to cccDNA. HBx protein controls transcription from HBV cccDNA by inducing ubiquitination of the Smc5/6 transcriptional suppressor complex, though this may not occur in other contexts, such as integrated HBV DNA [74]. We expand more on the as-yet hypothetical roles of HBV antigens expressed from the integrated form below in the “unanswered questions” section.

### 2.3. The Role of HBV Integration in HCC Initiation and Progression

The majority of integrated HBV DNA research has been focused on its potential to drive HCC. Indeed, the first descriptions of HBV DNA integrated into the host cell genome were of primary HCC tissues and HCC-derived cell lines, prompting suggestions that integrated HBV DNA was causative in tumorigenesis [46,68,69]. The reported mechanisms include (1) *cis*-mediated insertional mutagenesis of HCC-associated genes; (2) induction of chromosomal instability by integrated DNA; and (3) the expression of mutant HBV genes from the persistent integrated form. However, the mechanism of HBV-induced HCC carcinogenesis still remains unclear and poorly characterised [75].

#### 2.3.1. Insertional Mutagenesis

Studies on woodchuck hepatitis virus (WHV) demonstrated frequent integrations in (and subsequent transcriptional activation of) the Myc family of proto-oncogenes (in particular N-myc2) in woodchuck liver tumours [76,77,78]. As almost all woodchucks with chronic WHV infection develop HCC without cirrhosis within 4 years [79], WHV-associated HCC is proposed to be driven mostly by virus integration. However, studies of human HBV-associated HCC have shown more heterogeneity with less clear driving mechanisms.

Recent studies have used NGS technologies to find genetic characteristics of HBV-associated HCCs and characterise the preferential integration sites between tumour and matched non-tumour tissues [43,44,80,81,82,83]. Both greater numbers of integration events and increased integration frequency in coding or promoter regions have been detected in HCC compared to non-tumour tissue [43,81,84], though not in all studies [85]. In general, it is unclear whether these observations reflect HBV integration into these genomic areas as a driver for HCC, or are an outcome of tumour-initiating cells having greater susceptibility to integration in general (e.g., due to chromosomal instability or accumulation of deleterious passenger mutations associated with HCC progression) [86].

While integration occurs in random sites in the host cell genome [42,58,70,87], study of recurrent integration sites may show specific driver genes that cause clonal expansion of hepatocytes and outline possible mechanisms of hepatocarcinogenesis. Multiple studies have described recurrent integration into the telomerase reverse transcriptase (*TERT*) and *MLL4* genes accompanied by alteration in expression (likely driven by the Enhancer 2/Core promoter region of the integrated HBV DNA) [43,66,82,83,84,88,89]. Shiraishi et al. showed that *TERT* and *CDK15* contained integration breakpoints upstream of their respective transcriptional start sites, which generated fusion transcripts that were over-expressed [90]. In addition, while only slightly increasing expression in HCC samples, HBV-*MLL4* chimeric transcripts were out-of-frame, potentially leading to loss‑of‑function [90]. Other studies have shown highly increased transcription level in *MLL4* gene with the viral integration [80]. However, the proportion of HBV-associated HCCs containing integrations in (or near) *TERT* or *MLL4* occurs in a relatively low proportion (10–15%) of HCC [43,66]. Further, these changes only occur late in tumour development, suggesting a role in the progression (but not the initiation) of HCC [91].

HBV integrations can also lead to other (possibly oncogenic) virus–human transcript fusions found both in liver tissue and HBV-positive HCC cell lines [92]. HBV integration in host LINE results in the formation of the HBV–LINE1 fusion transcript that was reported in 23.3% of HCCs. This novel transcript was suggested to act as a long non-coding RNA that activates Wnt/β-catenin signalling, thereby promoting HCC [92]. However, HBV-LINE1 fusion transcript appears to be restricted to HBV genotype C in Asian ethnicity, and was not confirmed in HBV-related HCCs in European patients [93].

#### 2.3.2. Chromosomal Instability

HBV integration has been associated with genomic and chromosomal instability. HBV shows significant enrichment of integration near fragile sites, repetitive regions, CpG islands, and telomeres in tumours compared to non-tumour tissue [84,94]. Over half of the integration events were located in intergenic regions within the chromosomes of the human genome and in repeat sequences such as LINEs, short interspersed nuclear elements, or simple repeats (microsatellites) [94]. Furthermore, chromosomal rearrangements and copy number variations were associated with a large fraction of HBV-related HCCs [43,80], but this can be highly variable [95,96]. Integration into host scaffold/matrix attachment regions (S/MAR) has been observed in HCCs with chronic WHV infection [78,97] and human HCC cell lines [90], which may deregulate cellular oncoprotein expression and promote carcinogenesis [65,98,99]. However, the extent to which this occurs in a true in vivo HBV infection is currently unknown [85].

One of the limitations with NGS is the low coverage of HBV reads, leading to a loss of sensitivity. For example, Jiang et al. detected clonal expansion of hepatocytes carrying virus integration in the tumour samples but not in the matched non-tumour samples by NGS [80], however it has been established using more sensitive and specific techniques that clonal expansion occurs very early during chronic HBV infection in histologically-normal liver tissue [42,70]. Thus, the true spectrum of integrations in non-tumour tissue in NGS studies may not be complete, limiting interpretations.

#### 2.3.3. Expression of HBV Proteins with Oncogenic Potential from Integrated HBV DNA

Another possible mechanism by which integrated HBV DNA can drive HCC is through the generation and persistent expression of mutant HBV proteins. Expression of mutated or truncated HBV surface proteins (which are known to be produced by integrated HBV DNA) are associated with ER stress responses and may increase the risk of HCC [100,101,102]. Further, these mutant forms may give hepatocytes a proliferative advantage and have been shown to stimulate hepatocyte expansion [102,103,104,105]. Indeed, over-expression of these mutant forms induces precancerous liver lesions and HCC in animal models [106,107]. Moreover, overexpression of the C-terminal truncated HBx generated by integrated dslDNA forms has been described to induce stem-cell like properties [108], transformation and inhibition of apoptosis [109,110], and tumour invasion [111,112]. However, many physiologically-irrelevant phenotypes can infamously be generated by over‑expression of HBx and its mutants (as opposed to natural levels under its native promoter) [113]. As such, the contribution of these mutant proteins in a true chronic infection requires further research in appropriate and upcoming animal models.

In summary, the role of HBV integration in HCC initiation and progression is complex, and is likely to interact with many aspects in the liver microenvironment (e.g., cirrhosis and chronic inflammation). The lack of physiological models of HBV infection and a true HBV–HCC model that recapitulates these aspects makes it exceedingly difficult to classify and understand the contribution of HBV integration towards HBV-associated hepatocarcinogenesis.

## 3. Unanswered Questions

Given the limitations in small animal and cell culture models for true HBV infection, the body of knowledge related to HBV DNA integration remains incomplete. Below, we highlight several key questions that remain open in the field.

### 3.1. What Are the Dynamics of HBV DNA Integration throughout HBV Infection?

As mentioned previously, HBV DNA integration is likely to be an early event in individuals exposed to HBV. It is currently unclear exactly how early integration occurs during HBV infection, whether ongoing integration occurs throughout the course of a chronic HBV infection, or whether it exists as a stable equilibrium (with antiviral immune response targeting integrated forms as new integration events occur). If ongoing integration does occur through infection, it is likely to require either infection of novel hepatocytes or cycling of HBV dslDNA-containing nucleocapsids to the nucleus, with the rate of these phenomena in the HBV infected liver still not precisely known. The answers to these questions are likely to have implications on the development of potential therapies targeting HBV integration events.

### 3.2. How Does Integration Occur? What Cellular and Viral Factors Affect Integration?

**Figure 4:**
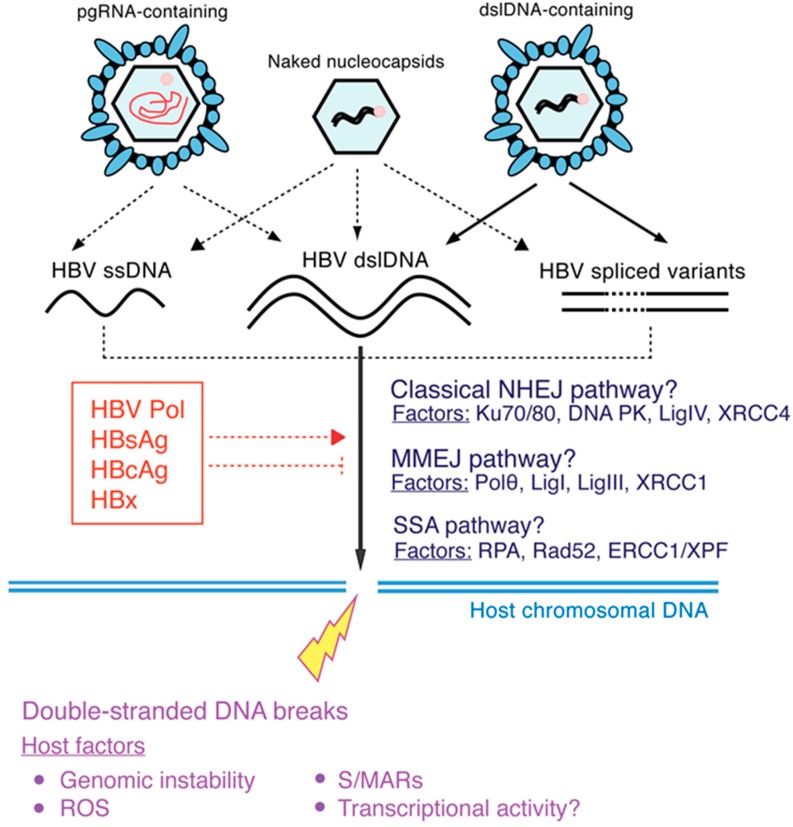
A model of potential molecular factors in HBV DNA integration. Various secreted HBV forms (**top row**) may be involved in bringing the potential molecular substrates for HBV DNA integration (**black**) into the cell. Both reported (**solid arrows**) and as-yet purely hypothetical pathways (**dashed arrows**) for HBV DNA integration into the host cell genome (**light blue double lines**) via potential cellular double-stranded DNA (dsDNA) repair mechanisms (**dark blue**) are shown, depicted with the key host enzymes involved. The substrates for integration include full-length HBV dslDNA, though the HBV single-stranded DNA (ssDNA) and spliced variant dsDNA forms may also contribute. Further, it is currently unknown whether viral proteins (**red**) play either an inhibitory or inducing role in HBV DNA integration. Integration occurs at dsDNA breaks in the host cell genome, and relevant host factors that induce these breaks are shown in purple. MMEJ, microhomology‑mediated end-joining; NHEJ, non-homologous end-joining; SSA, single-stranded annealing; ROS, reactive oxygen species; S/MARs, scaffold/matrix attachment regions.

While HBV DNA integration is likely to be mediated by enzymes involved in the cellular DNA repair pathways (Figure 4), it is unknown which particular pathway(s) is/are active in the process. Several dsDNA break repair pathways have been described, each involving both unique and shared host enzymes [45,114,115]. A thorough understanding of which repair mechanisms are involved could reveal the (possibly pathogenic) properties of cells containing HBV integrations. For example, hepatocytes with greater genomic instability (caused by dysfunction in particular DNA repair pathways) may be more likely to contain integrations, and the clonal expansion of such hepatocytes could be a marker of HCC risk.

Further, it is unknown which other cellular proteins may have an important role. Potential factors may include: those that promote nuclear dslDNA levels (e.g., by increasing priming from the DR1 region, inducing trafficking of mature nucleocapsids to the nucleus, or inhibiting dslDNA circularisation into cccDNA); those that bind to DNA to mediate cell repair mechanisms; or those that alter chromosomal modelling and stability.

Similarly, there is a poor understanding of viral factors that affect integration. For example, it is unknown whether viral proteins are involved in the process of integration. The HBV polymerase, core, and X proteins are all potential candidates for direct involvement in the integration process due to their DNA binding activities. The role of HBV genotype in integration also remains an open question. In addition, the form of HBV that enters a hepatocyte may alter the localisation of its DNA or RNA contents. Minor forms mentioned above—such as naked nucleocapsids containing HBV DNA or pgRNA-containing viruses—may play a role in delivering HBV DNA to the nucleus whereupon integration occurs.

Finally, while dslDNA is the presumed form that undergoes HBV integration, HBV single‑stranded DNA (ssDNA) replicative intermediates or HBV spliced variants could also play a role (Figure 4), as these forms possess the same terminal DNA sequences as dslDNA. In particular, almost all HBV spliced variants are present as dslDNA forms due to the loss of *cis*-acting circularisation signals [116], making them a likely substrate for HBV DNA integration.

While these pathways remain currently uncharacterised, we have here outlined working falsifiable hypotheses that can be tested in future in vitro studies. A better understanding of the molecular factors involved in HBV DNA integration is key in shedding light on the roles that these persistent forms play and in developing potential therapeutics against ongoing integration.

### 3.3. To What Extent Does Integration Contribute to HBV Replication and Persistence?

HBV proteins expressed from the integrated form may allow cccDNA-independent expression and persistence of viral antigens. After clonal expansion of hepatocytes, an estimated 1% of hepatocytes harbor detectable integration events [42]. Even if only a minority of integrated HBV DNA express wild-type subgenomic transcripts, these hepatocyte clones could form persistent reservoirs for HBV antigen expression. If these hepatocytes become infected by otherwise replication-incompetent forms (e.g., HBV spliced variants), HBV proteins could be supplied in trans by integrated forms to support viral replication.

Additionally, integrated forms may also play a role in escaping the immune response via modulation of HBV transcription. Inhibition of HBV protein expression (e.g., using shRNA-based knock-down) induces a recovery of an anti-viral adaptive immune response in the immune-competent mouse models [117,118,119]. Integrated forms are known to express both wild-type and mutant versions of the HBsAg. Integrated HBV DNA could be a mechanism for persistence by maintaining liver expression of HBsAg, while not producing HBeAg that is targeted by the anti-viral immune response. In this speculative model, as the anti-HBe antiviral response intensifies, clonal expansion of hepatocytes would increase the number of cells containing integrated HBV that express immunosuppressive HBsAg proteins, thereby creating a new equilibrium where HBV is still persistent within the liver.

While HBx mutations are common in integrated HBV forms within the chronically infected liver and tumours [120,121], functional HBx may also be expressed from integrated forms in some hepatocytes. As mentioned above, expression of HBx from integrated HBV DNA forms are likely to be differentially regulated compared to cccDNA. Thus, integrated forms would provide variation in HBx-regulated HBV expression, such that (under the evolutionary pressure of the antiviral response) HBV antigen-expression levels below the threshold of immune-mediated cell death were selected for. Moreover, expression from transcriptionally-silent cccDNA could be kick-started by the HBx expressed from integrated forms. Considered together, integrated HBV DNA forms could form an elegant pathway for the HBV genome to control expression of HBV mRNA under different host-imposed conditions.

### 3.4. Do Integrated Forms Persisting after Functional Cure of CHB Contribute to HCC Risk?

It is unknown whether these persistent integrated forms contribute to HCC risk, which is reduced but not completely eliminated in functionally-cured HBV patients in the HBsAg-negative phase (so called “occult HBV infection”, in which patients are HBsAg-negative, but have detectable intrahepatic HBV DNA) [122,123]. Animal model experiments suggest that integrated forms of HBV persist after the resolution of infection [38,39,41]. In patients, persistent integrated forms have been detected in occult HBV infection [124], and may act as targets for low-level ongoing antiviral immune attack. As occult HBV infection has been associated with increased risk of HCC and greater severity of liver disease [122,123], these integrated forms are relevant to the clinical management of infected individuals.

However, the carcinogenic contribution of integrated HBV DNA is difficult to separate from the contribution of the inflammatory and cirrhotic liver microenvironment, so the causal link between HBV DNA integration and HCC is still unclear. As integration occurs early while HCC generally occurs late, the direct mechanism of HBV DNA integration in hepatocarcinogenesis is weak, requires other co-factors (e.g., chronic inflammation), and/or acts slowly. This makes studying the carcinogenic role of HBV DNA integration in physiologically-relevant conditions particularly challenging.

### 3.5. Can Integrated HBV DNA Contribute to the Replication of Hepatitis Delta Virus (HDV)?

HDV is a satellite virus of HBV [125]. Chronic co-infection with HDV is associated with worse clinical prognosis: a two-fold increased rate of developing cirrhosis and three-fold higher risk of HCC development (compared to HBV-monoinfected patients) [126,127]. HDV requires the large and the small HBV envelope protein for the production of new virions and dissemination. While HBsAg antigen expressed from integrated HBV DNA can support HDV assembly and release in vitro [128], the possibility, extent, and impact of this phenomenon occurring in a true in vivo chronic co-infection remains unknown. However, from an experimental point of view, nothing argues against the possibility that HDV can replicate in hepatocytes devoid of HBV taking advantage of HBsAg produced from integrated HBV DNA. The presence of hepatocytes that have the heritable ability to support HDV replication but are HBV replication-deficient would impact our understanding of HDV persistence and strategies in developing anti-HDV therapies.

## 4. Conclusions

With the scant available knowledge of its molecular mechanisms, roles in virus replication, and clinical implications, HBV DNA integration remains an intriguing phenomenon that still requires intense study. Particular focus should be placed on integration and its involvement in HBV replication and persistence, which underpins our understanding of how to develop a cure for CHB patients. Much of our understanding has been extrapolated from animal models, but many important unanswered questions on the role of HBV integration (especially with respect to its role in HBV chronicity and HCC development) remain and may be addressed using recently-developed, physiologically-relevant cell culture and in vivo infection models, such as humanized mice. Of course, these models must ultimately provide physiological relevance and accuracy. As such, studies based on human liver tissue samples are indispensable to gain insights about the biological and clinical significance of HBV integration. While the exact function of HBV DNA integration is unknown at this time, further investigation of this feature that is shared by all known members of the *Hepadnaviridae* will undoubtedly improve our understanding of the persistence, progression, and pathogenesis of chronic HBV infections.
